# Size Engineering of Ti_3_C_2_T_x_ Nanosheets for Enhanced Supercapacitance Performance

**DOI:** 10.3390/molecules30020241

**Published:** 2025-01-09

**Authors:** Haosheng Liu, Xin Chang, Lu Li, Mingyi Zhang

**Affiliations:** Key Laboratory for Photonic and Electronic Bandgap Materials, Ministry of Education, School of Physics and Electronic Engineering, Harbin Normal University, Harbin 150025, China; liuhaoshengbb@icloud.com (H.L.); cx20220718@163.com (X.C.)

**Keywords:** Ti_3_C_2_T_x_, ultrasonic, supercapacitance, filtration, nanosheets

## Abstract

In this research, we synthesized a series of Ti_3_C_2_T_x_ nanosheets with varying lateral dimensions and conducted a thorough investigation into the profound relationship between the electrochemical performance of Ti_3_C_2_T_x_ materials and their lateral sizes. This study innovatively incorporates a clever combination of small-sized and large-sized Ti_3_C_2_T_x_ nanosheets in the electrode preparation process. This strategy yields excellent results at low scan rates, with the fabricated electrode achieving a high volumetric capacitance of approximately 658 F/g. Even more remarkable is the fact that, even under extreme testing conditions where the scan rate surges to 10 V s^−1^, the electrode retains its capacitive characteristics robustly without any significant performance degradation. These outstanding characteristics underscore the exceptional ability of Ti_3_C_2_T_x_ electrode materials to maintain high energy storage capacity during rapid charge–discharge cycles, holding significant importance for advancing the development of electrochemical energy storage devices with fast response times and high power densities.

## 1. Introduction

Energy storage devices are facing unprecedented challenges: they need to provide higher energy density and power density over longer operating cycles. To enhance these critical performance indicators, we must not only discover new materials with exceptional energy storage potential, but also deeply understand how to optimize the assembly of these materials into electrode structures with excellent ion and electron conductivity [1,2,3]. In recent years, a class of two-dimensional (2D) materials known as MXene has stood out due to their unique properties and has become one of the preferred electrode materials for energy storage devices. MXene, a family composed of transition metal carbides, nitrides, and carbonitrides, can be generally expressed by the formula M_n+1_X_n_T_x_. Here, M represents early transition metal elements, X represents carbon or nitrogen, and T_x_ refers to surface functional groups attached to the material, such as hydroxyl (OH), oxygen (O), and fluorine (F). Since the discovery of the first MXene material, Ti_3_C_2_T_x_, in 2011, these materials have quickly garnered widespread attention from the scientific research community and have shown broad application prospects in various fields such as energy storage, water desalination, catalytic reactions, and electromagnetic shielding [4,5,6,7,8].

Particularly noteworthy is the electrochemical performance of MXene in the field of supercapacitor electrodes, which not only holds value for theoretical research but also plays a pivotal role in practical applications [9,10]. The charge storage mechanism of MXene is primarily based on pseudocapacitance, which is fundamentally different from the traditional electric double-layer capacitance (EDLC) mechanism relied upon by carbon materials such as graphene. In contrast, MXene materials store charge through rapid surface redox reactions, a mechanism that enables them to provide extremely high specific capacitance values, like other well-known pseudocapacitive materials such as MnO_2_ and RuO_2_. Furthermore, the MXene family possesses two major advantages: high electrical conductivity and highly accessible interlayer spacing. These characteristics enable such materials to maintain stable performance during high-rate charging and discharging processes and exhibit exceptional high-power density properties [11,12,13]. These attributes undoubtedly lay a solid foundation for the application of MXene in energy storage devices such as supercapacitors and open broad prospects for such materials to play a more important role in the energy field in the future.

The reason why Ti_3_C_2_T_x_ material exhibits exceptional capacitance performance lies fundamentally in its unique layered structure, which is formed by three layers of titanium atoms tightly sandwiching two layers of carbon atoms, creating a stable and efficient electron transport channel. The high pseudocapacitance characteristics of Ti_3_C_2_T_x_ benefit from the functionalized transition metal layer on its surface, which not only increases the active sites of the material but also facilitates rapid charge adsorption and desorption processes, thereby significantly enhancing its capacitance performance [14].

However, it is worth noting that in past research endeavors, scientists have tended to focus more on the chemical composition and structural characteristics of Ti_3_C_2_T_x_ and other two-dimensional MXene materials, while failing to give sufficient attention to the critical factor of the lateral size of MXene sheets [15]. In fact, the size of MXene sheets has a significant impact on their electrochemical performance. This is because the synthesis method and subsequent processing conditions greatly determine the final layer size of the product [16]. MXene sheets of different sizes exhibit notable differences in terms of ion diffusion paths, charge storage capacity, and stability of electrode materials. Unfortunately, current research on the size-dependent properties of MXene are relatively scarce, which to some extent limits our comprehensive and in-depth understanding of their electrochemical performance and the formulation of optimization strategies.

**Figure 1:**
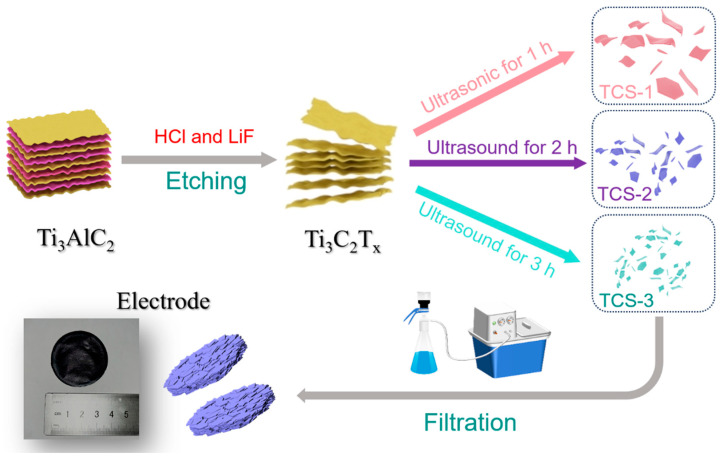
Schematic illustration of the synthesis process of Ti_3_C_2_T_x_ nanosheets.

In this study, we successfully employed ultrasonic fragmentation technology to meticulously treat Ti_3_C_2_T_x_ material through ultrasonic processing, thereby obtaining a series of Ti_3_C_2_T_x_ nanosheets with precisely controlled sizes. This achievement is illustrated in Figure 1. This method is not only straightforward in operation but also ensures uniform distribution of Ti_3_C_2_T_x_ nanosheets within the electrode, thereby maximizing their electrochemical performance. Through in-depth experimental analysis and performance testing, we revealed the importance of controlling the size of synthesized two-dimensional Ti_3_C_2_T_x_ nanosheets for designing electrodes with high ionic and electrical pseudocapacitance. In exploring their application in the field of supercapacitors, we found that smaller-sized Ti_3_C_2_T_x_ nanosheets exhibited remarkable electrochemical performance in sulfuric acid electrolyte, with a specific capacitance as high as 658 F/g. These data fully demonstrate the enormous potential of small-sized Ti_3_C_2_T_x_ in energy storage. More importantly, after undergoing up to 20,000 charge–discharge cycles, these Ti_3_C_2_T_x_ nanosheets maintained satisfactory cycle stability and rate performance, further validating their reliability and durability in practical applications.

## 2. Results and Discussion

**Figure 2:**
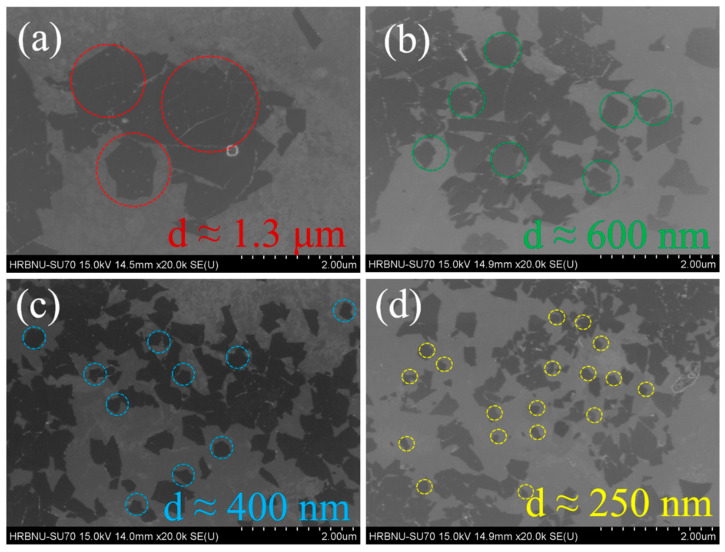
SEM images of (**a**) TCS-0; (**b**) TCS-1; (**c**) TCS-2; (**d**) TCS-3.

The SEM images presented in Figure 2 show Ti_3_C_2_T_x_ nanosheets obtained without ultrasonic treatment and after undergoing ultrasonic treatment for different durations. These images vividly reveal the significant impact of ultrasonic treatment on the morphological structure of Ti_3_C_2_T_x_ nanosheets. Firstly, we observe that all four samples exhibit typical two-dimensional layered structural characteristics. In particular, the Ti_3_C_2_Tx nanosheets without ultrasonic treatment (Figure 2a), which were prepared through an etching process, have relatively large sizes with an average diameter of approximately 1.3 μm. However, significant changes occur when ultrasonic treatment is introduced. As shown in Figure 2b–d, with the gradual increase in ultrasonic treatment time from 1 h to 3 h, the size of the Ti_3_C_2_T_x_ nanosheets undergoes a noticeable reduction process. Specifically, the size of the nanosheets gradually decreases from an initial approximate size of 600 nanometers to a final size of about 250 nanometers. This change intuitively demonstrates the “cutting” effect of ultrasound at the nanoscale, where energy generated by high-frequency vibrations effectively breaks down larger nanosheets into smaller ones [17,18]. It is worth noting that ultrasonic treatment not only alters the size of the nanosheets but may also have a profound impact on their surface structure and properties. The reduction in size may lead to rearrangement or detachment of functional groups on the nanosheet surface, thereby changing their catalytic, adsorption, and other properties [19,20].

**Figure 3:**
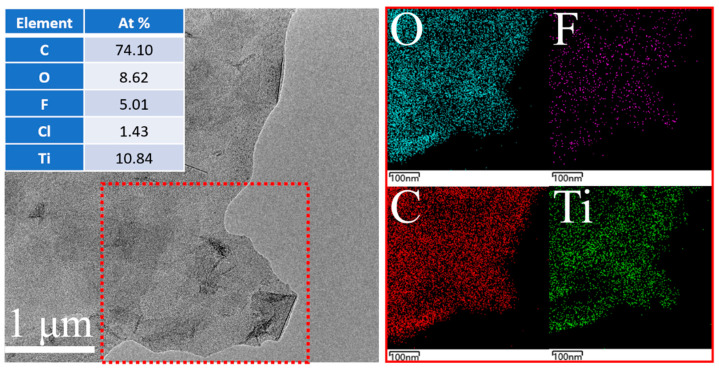
TEM images, elemental percentage distribution, and elemental mappings of TCS-3.

The image presented in Figure 3 showcases the Transmission Electron Microscope (TEM) imagery of the TCS-3 sample, a high-precision imaging technique that profoundly reveals the remarkable microscopic structural characteristics of the sample. In the imagery, the TCS-3 sample stands out with its exceptional two-dimensional sheet-like morphology, which typically indicates a higher specific surface area and potentially superior performance in the field of nanomaterials. Particularly noteworthy is that the ultrasonic treatment process has finely adjusted the size of the nanosheets with almost no visible damage or impact on their surfaces, with no signs of cavities or other secondary structures in the image. This strongly demonstrates the gentle regulation and precise operation capabilities of ultrasonic treatment in this context. Further investigation through mapping energy spectrum analysis reveals that elements such as C (carbon), O (oxygen), Ti (titanium), and F (fluorine) are uniformly and consistently distributed across the nanosheets. This distribution pattern not only greatly enhances the structural stability of the material but also likely has a profound positive impact on its physicochemical properties. When we specifically focus on the atomic ratio of the elements, it is clear that the atomic ratio of C, O, and Ti reaches 74.1:8.62:10.84. In this ratio, the content of C is significantly higher than that of the traditional Ti_3_C_2_T_x_ nanomaterial composition. This phenomenon is mainly due to the interference effect of the carbon film in the copper grid during the testing process, leading to an elevated C element content.

**Figure 4:**
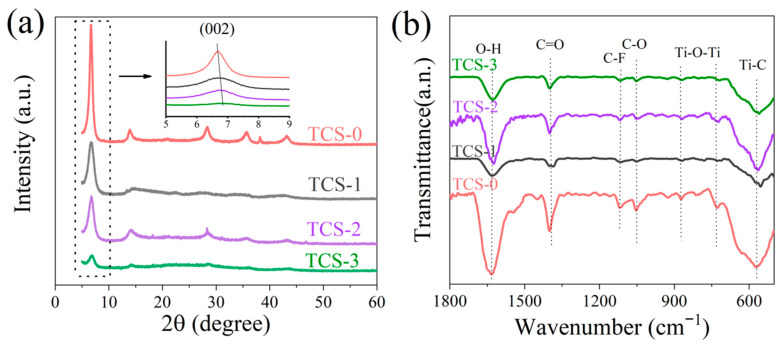
(**a**) XRD patterns and (**b**) FTIR spectra of TCS-0, TCS-1, TCS-2, and TCS-3.

Figure 4 visually presents a comparison of the X-ray diffraction (XRD) patterns for the four samples: TCS-0, TCS-1, TCS-2, and TCS-3. In the XRD patterns of all samples, a typical Ti_3_C_2_T_x_ (002) characteristic peak located in the low-angle region can be observed, which is a hallmark of the MXene material Ti_3_C_2_Tx. However, it is noteworthy that, compared to the TCS-0 electrode without ultrasonic treatment, TCS-1, TCS-2, and TCS-3 electrodes that underwent ultrasonic treatment for different durations exhibited significant changes in the position of the (002) peak. Specifically, as the duration of ultrasonic treatment gradually increased, the (002) peaks of these three samples shifted towards higher angles, accompanied by an increase in the full width at half maximum (FWHM) [21]. This phenomenon not only reveals the significant impact of ultrasonic treatment on the material size, namely reducing the size of Ti_3_C_2_T_x_ sheets, but also indicates that ultrasonic treatment profoundly alters the crystal structure of Ti_3_C_2_T_x_. The changes in crystal structure induced by ultrasonic treatment are specifically manifested as potential adjustments in interlayer spacing and a certain degree of reduction in in-layer orderliness, which provides more favorable conditions for the intercalation of small molecules such as H_2_O [22]. Smaller sheet structures imply widened interlayer channels and more unobstructed pathways, thereby facilitating the effective penetration and intercalation of small molecules like H_2_O within the Ti_3_C_2_T_x_ layers [23]. Therefore, ultrasonic treatment not only optimizes the size distribution of Ti_3_C_2_T_x_ materials but also enhances their potential performance in applications involving small-molecule intercalation and transport by regulating their crystal structure. In the Fourier Transform Infrared (FTIR) spectra of the four samples TCS-0, TCS-1, TCS-2, and TCS-3 (Figure 4b), the characteristic absorption peaks are clearly visible: Specifically, the absorption peak located at approximately 1051 cm^−1^ corresponds to the vibrational mode of the C-O bond, while the absorption peak near 1090 cm^−1^ definitely indicates the presence of the C-F bond. The absorption peak at around 1650 cm^−1^ is closely related to the vibration of the -OH group. Additionally, at the position of about 665 cm^−1^ in the spectrum, we observe an absorption peak that originates from the vibration of the Ti-O bond. These meticulous observations strongly suggest that despite differences in sample size, there is no significant impact on the types of functional groups present on the surface of the Ti_3_C_2_ material.

**Figure 5:**
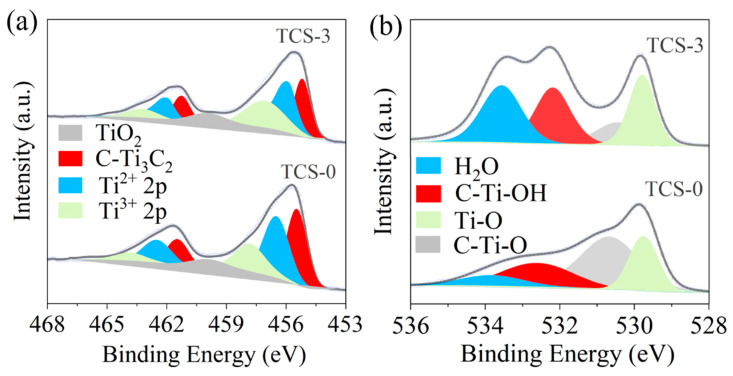
High-resolution XPS spectra: (**a**) O 1s, and (**b**) Ti 2p.

To delve deeper into the surface chemical composition and valence state characteristics of Ti_3_C_2_T_x_ films (represented by TCS-0 for untreated samples and TCS-3 for samples sonicated for 3 h), we employed advanced X-ray Photoelectron Spectroscopy (XPS) analysis techniques. As shown in Figure 5a, in TCS-0 films, the characteristic peaks for Ti^2+^ 2p3/2 and Ti^3+^ 2p3/2 are located at 456.1 eV and 457.2 eV, respectively. However, when the Ti_3_C_2_T_x_ films undergo sonication treatment, transitioning to a more activated state, these peaks undergo subtle but significant shifts, specifically with the Ti^2+^ 2p3/2 peak moving to 455.3 eV and the Ti^3+^ 2p3/2 peak shifting to 456.6 eV. This change is attributed to the firm adsorption of hydrated protons (H_3_O^+^) onto the surface of Ti_3_C_2_T_x_ through the formation of hydrogen bonds, a process that effectively modulates the electron cloud density of Ti atoms and enhances their electrochemical activity [24,25].

Furthermore, through detailed analysis of high-resolution O 1s spectra (Figure 5b), we can clearly observe four distinct oxygen bonding states: Ti-O (located at 530.2 eV), C-Ti-O (located at 531.4 eV), C-Ti-OH (located at 532.7 eV), and H_2_O (located at 533.8 eV). Notably, after 3 h of sonication treatment, the binding energies of these oxygen bonds in TCS-3 samples exhibit a trend of shifting to lower values compared to TCS-0. This phenomenon reveals that sonication treatment not only promotes the extensive penetration of hydrated protons but also leads to significant changes in the electron cloud density within Ti_3_C_2_T_x_. This redistribution of electron density is crucial for accelerating the hopping transport of protons between Ti_3_C_2_T_x_ layers, thereby enhancing its proton conduction performance [26,27,28].

Particularly noteworthy is the higher C-Ti-OH content in TCS-3 compared to TCS-0, as evidenced by the intensity comparison between C-Ti-OH and C-Ti-O. This discovery has multiple implications: Firstly, it suggests that the desorption of hydrogen atoms from surface -OH groups is a key step in the energy storage process of Ti_3_C_2_T_x_. If the positive potential of Ti_3_C_2_T_x_ can be increased without triggering the decomposition of the aqueous electrolyte, more hydrogen atoms will be released from -OH functional groups, significantly enhancing the charge storage capacity of Ti_3_C_2_T_x_ [23]. Secondly, the presence of -OH groups is crucial for the effective intercalation of H_2_O molecules between Ti_3_C_2_T_x_ layers, facilitating the penetration and distribution of water molecules and contributing to the enhancement of the material’s electrochemical activity [23,29]. Lastly, the increase in -OH groups also significantly improves the hydrophilicity of Ti_3_C_2_T_x_, making it more compatible with aqueous environments and favorable for the expression of electrochemical performance [30]. Additionally, the significant increase in the H_2_O peak in TCS-3 directly reflects a substantial elevation in its bound water content. This change not only confirms the extensive binding of hydrated protons to Ti_3_C_2_T_x_ but also indicates the formation of more abundant and stable hydrated structures on the surface and within Ti_3_C_2_T_x_ [31,32].

**Figure 6:**
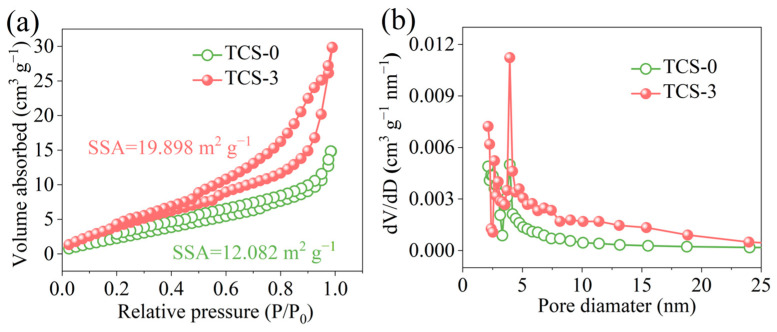
(**a**) N_2_ adsorption–desorption isotherms and (**b**) pore size distributions of samples TCS-0 and TCS-3.

The specific surface area and pore size distribution of samples TCS-0 and TCS-3 were analyzed through N_2_ isothermal adsorption and desorption measurements (Figure 6a,b). The results indicate that sample TCS-0 exhibits typical type-IV isotherms, which are characteristic of mesoporous materials. The specific surface area of sample NMO was measured to be 12.082 m^2^/g. After the ultrasonic treatment, the specific surface area of the resulting obtained NMC-2 sample is notably larger, reaching 19.898 m^2^/g. The center of the pore size distribution center of sample NMC-2 is 4.81 nm; thus, this sample has a mesoporous structure, which facilitates rapid ion migration and diffusion. The vast open spaces provide a larger contact area between the active material and the electrolyte, while the high specific surface area provides numerous electroactive sites, which enhances both ion and electron transfer, consequently improving the capacitive performance of the material.

**Figure 7:**
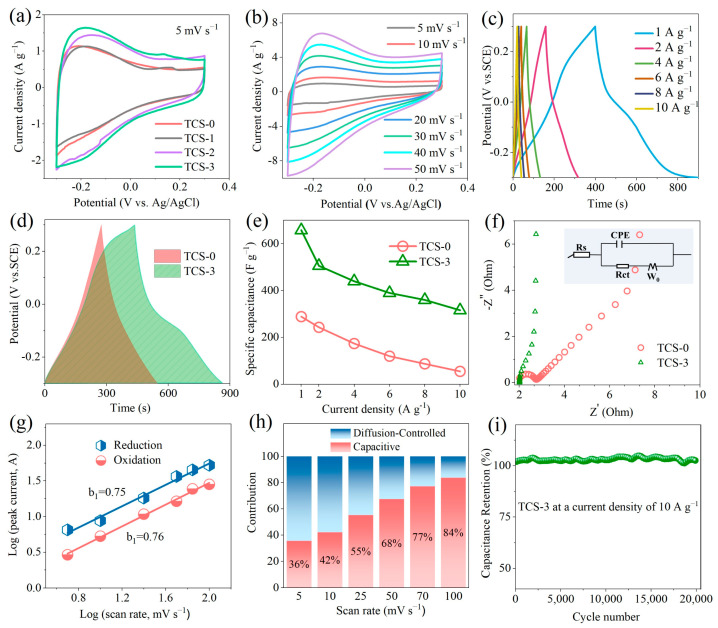
(**a**) Cyclic voltammetry curves of Ti_3_C_2_T_x_ at 5 mV s^−1^ after different ultrasound durations, (**b**) cyclic voltammetry curves of TCS-3 at scan rates ranging from 5 to 50 mV s^−1^, (**c**) galvanostatic discharge curves of TCS-3 at current densities ranging from 1 to 10 A g^−1^, (**d**) galvanostatic discharge curves of TCS-0 and TCS-3 at a current density of 1 A g^−1^, (**e**) specific capacitance at various current densities, (**f**) Nyquist plots of TCS-0 and TCS-3, (**g**) relationship between peak current and scan rate for TCS-3, (**h**) capacitance contributions of surface-controlled and diffusion-controlled processes at different scan rates for TCS-3, and (**i**) cycling performance of TCS-3 at a current density of 10 A g^−1^.

The electrochemical properties of the samples were thoroughly investigated in a standard three-electrode system using an H_2_SO_4_ aqueous solution as the electrolyte. Figure 7a visually presents a comparison of the cyclic voltammetry (CV) curves for TCS-0, TCS-1, TCS-2, and TCS-3 at a scan rate of 5 mV s^−1^. From the figure, it can be observed that although the Ti_3_C_2_T_x_ membrane electrodes of various sizes exhibit similar redox peaks and CV curve shapes, the redox peaks of TCS-3 are particularly prominent, suggesting it may possess superior electrochemical activity. Furthermore, Figure 7b details the changes in the CV curves of the TCS-3 electrode at different scan rates ranging from 5 to 50 mV s^−1^, with the potential window set to −0.3 to 0.3 V (relative to the Ag/AgCl reference electrode). Notably, the CV curves exhibit clear redox peaks at approximately −0.25 V, which are attributed to the proton intercalation and deintercalation processes during charging and discharging of Ti_3_C_2_T_x_ in an acidic medium [22]. As the scan rate gradually increases, despite slight changes in the redox peak potentials and CV curve shapes, TCS-3 maintains good rate performance, demonstrating the robustness of its electrochemical process.

To gain a deeper understanding of the electrochemical energy storage characteristics of TCS-3, Figure 7c shows its galvanostatic charge–discharge (GCD) curves at different current densities. The GCD curves exhibit nearly symmetric nonlinear features, which not only indicate that the TCS-3 electrode exhibits typical pseudocapacitive behavior, consistent with the analysis of the CV curves, but also reveal its high Coulombic efficiency. By comparing the GCD curves of TCS-0 and TCS-3 at a current density of 1 A g^−1^ (Figure 7d), it can be clearly seen that the TCS-3 film has the longest discharge time, thus possessing the largest specific capacitance. Specifically, at a current density of 1 A g^−1^, the specific capacitances of TCS-0 and TCS-3 are 289 F g^−1^ and 658 F g^−1^, respectively, and TCS-3 has a lower voltage drop, mainly due to its smaller internal resistance [33]. Samples TCS-1 and TCS-2 ought to be assessed under a range of different current densities and scan rates as well (Figure S1). Figure 7e compares the specific capacitance performance of TCS-0 and TCS-3 at different current densities in detail. As the current density increases, the specific capacitances of both decrease, but TCS-3 experiences a significantly smaller decrease, demonstrating superior rate capability. At high current densities of 2 A g^−1^and 10 A g^−1^, the specific capacitances of TCS-0 and TCS-3 are 243 F g^−1^ and 506 F g^−1^, and 87 F g^−1^ and 316 F g^−1^, respectively, with corresponding capacitance retention rates of 30% and 48%. This result further validates the excellent rate performance of TCS-3, which is superior to previously reported MXene films, mainly due to its unique mesoporous structure and increased interlayer spacing [34]. To delve into the charge transfer resistance characteristics of the electrodes, electrochemical impedance spectroscopy (EIS) tests were conducted. Figure 7f compares the EIS data for TCS-0 and TCS-3. In the high-frequency region, TCS-3 exhibits lower impedance, which may be due to the presence of more hydrated protons on its surface and the preservation of the integrity of the nanosheets during mild ultrasonic treatment, thereby reducing ohmic resistance. In the low-frequency region, TCS-3 has a steeper slope, indicating faster ion diffusion rates [29]. To gain insight into the charge storage mechanism, the power law equation (i = av^b^) was used to calculate the relationship between the peak current of the CV curves and the scan rate at different scan rates, yielding b-values [35,36]. Figure 7g shows the log(v) − log(i) plots for TCS-0 and TCS-3 in the range of 5 to 100 mV s^−1^. The b-values for the anode and cathode peaks of TCS-3 are 0.75 and 0.76, respectively, which are higher than the 0.69 and 0.70 for TCS-0, respectively, indicating that surface-controlled processes dominate during charging and discharging, thereby giving TCS-3 excellent rate performance. Additionally, by quantifying the contributions of surface-controlled and diffusion-controlled processes (i(V) = k_1_v + k_2_v^−1/2^) [36], Figure 7h reveals that as the scan rate increases, the proportion of surface-controlled capacitance increases, while diffusion-controlled capacitance decreases accordingly. At a scan rate of 100 mV s^−1^, the surface-controlled capacitance contribution of TCS-3 is as high as 84% and is almost unaffected by the scan rate, which further confirms the significant improvement in ion transport pathways due to its increased interlayer spacing and mesoporous structure. Furthermore, the surface-controlled and diffusion-controlled capacitance contributions for TCS-0, TCS-1, and TCS-2 are presented in Figure S2 [37,38,39,40]. Finally, Figure 7i shows the capacitance retention rate of TCS-3 after 20,000 charge–discharge cycles, indicating that it still retains about 100% of its initial capacitance, fully demonstrating that TCS-3, like Ti_3_C_2_T_x_, possesses excellent cycle stability. The primary reason for this performance enhancement is the unique advantages possessed by the smaller nanosheets, resulting in the TCS-3 exhibiting a significantly higher value compared to the reported MXene nanosheets (Table S1).

## 3. Experimental Section

### 3.1. Materials

HCl and LiF were purchased from Zhiyuan Reagent (Tianjin, China). Ti_3_AlC_2_ powder were purchased from Xianfeng Nanomaterial Technology Co., Ltd. (Nanjing, China). Analytical-grade chemicals that had not been further purified were used in this study.

### 3.2. Synthesis of Ti_3_C_2_T_x_ Nanosheets

In a dedicated Teflon beaker, 20 mL of HCl solution was first accurately measured and 1.56 g of LiF powder was added. Subsequently, a stirrer was used to thoroughly mix the mixture until the LiF was completely dissolved in the HCl. Next, in a gradual and controlled manner, 1 g of Ti_3_AlC_2_ powder was slowly added to the beaker with continuously stirring. This step requires particular caution to prevent the powder from being added too quickly, which could lead to too vigorous a reaction. The Teflon beaker containing the reaction mixture was placed into a constant-temperature water bath pot, with the temperature set to 38 °C, and under continuous stirring for 48 h at this temperature. This step aims to allow HCl and LiF to fully react with Ti_3_C_2_T_x_, thereby exfoliating out Ti_3_C_2_T_x_ nanosheets. After the reaction was completed, the obtained sample was centrifuged and washed with 1 M HCl solution and 1 M LiCl solution, respectively, at 8000 revolutions per minute, with each solution being used for three washes. This step removes residual HCl, LiF, and possibly generated byproducts. Subsequently, the sample was further centrifuged and washed repeatedly with deionized water until the pH value of the supernatant after centrifugation approached the target value. At this point, a dark green Ti_3_C_2_T_x_ suspension was observed in the centrifuge tube. This indicates that Ti_3_C_2_T_x_ nanosheets were successfully exfoliated from Ti_3_AlC_2_. Finally, the upper suspension was carefully collected and stored in a refrigerator at 4 °C. Thus, we successfully prepared Ti_3_C_2_T_x_ nanosheets, providing an important material basis for their subsequent research and applications.

Next, we carefully poured the prepared Ti_3_C_2_T_x_ nanosheet colloidal solution into a sample bottle. Then, argon gas (Ar) was purged into the bottle for 15 min to ensure a stable environment inside. Afterward, the sample bottle cap was quickly tightened, and sealing film was used to securely encapsulate it, preventing any gas leakage. Subsequently, the entire sealed sample bottle was placed into an ultrasonic cleaning machine, and the ultrasonic treatment process was initiated.

We employed this method to subject the samples to ultrasonic treatment for different durations: 1 h, 2 h, and 3 h; after the treatment was completed, the samples from these three different time points were named TCS-1, TCS-2, and TCS-3, respectively. For comparison, the Ti_3_C_2_T_x_ nanosheets that did not undergo ultrasonic treatment were named TCS-0.

### 3.3. Characterization

The phase of the samples was analyzed using an X-ray diffractometer (XRD, D/max-2600/pc, Rigaku, Tokyo, Japan). Subsequently, the morphology and microstructure of the samples were examined through scanning electron microscopy (SEM, Hitachi SU70, Tokyo, Japan). The internal structure was analyzed using transmission electron microscopy and high-resolution transmission electron microscopy (TEM, Tecnai F20, FEI, Hillsboro, OR, USA). Furthermore, the composition of the samples, the valence states of each element within the component, and their bonding states were analyzed using an X-ray photoelectron spectrometer (XPS, AXIS Supra+, Shimadzu, Kyoto, Japan).

### 3.4. Electrochemical Measurements

Using a vacuum filtration technique, we successfully prepared approximately 30 mg of Ti_3_C_2_T_x_ thin film from colloidal solutions of Ti_3_C_2_T_x_ with different sizes, utilizing a polytetrafluoroethylene (PTFE) microfiltration membrane with a diameter of 50 mm and a pore size of 0.1 μm as the filtration medium. Subsequently, this Ti_3_C_2_T_x_ thin film was precisely cut into small pieces with an area of 1/4 of a square centimeter (weighing approximately 0.4 mg), which served as the working electrode. Meanwhile, a carbon rod was selected as the counter electrode, and Ag/AgCl was used as the reference electrode, together forming a three-electrode testing system.

The electrochemical performance measurements were conducted using the VMP3 electrochemical workstation manufactured by Biologic in Grenoble, France. During the cyclic voltammetry (CV) tests, measurements were taken within a potential range of −0.3 V to +0.3 V at scan rates ranging from 5 to 50 mV/s. For the galvanostatic charge–discharge (GCD) tests, measurements were conducted at current densities ranging from 1 A/g to 10 A/g, with a special emphasis on conducting 20,000 charge–discharge cycles at a current density of 10 A/g to assess its cycling stability. Additionally, electrochemical impedance spectroscopy (EIS) tests were performed at open-circuit potential, with an amplitude set at 5 mV and a frequency range spanning from 10 mHz to 100 kHz. During these electrochemical tests, we paid particular attention to ensuring that the mass loading of the thin film was as uniform as possible, thereby minimizing the potential negative impact of material thickness on capacitance performance.

The specific capacitance of the samples was calculated using the following formula:

(1) $$C=\frac{I\times \Delta t}{m\times \Delta V}$$

where *C*, Δ*V*, *I*, *m*, and Δ*t* represent the specific capacitance, potential window, discharge current, mass of the active material, and discharge time, respectively [17].

## 4. Conclusions

In summary, we successfully employed a straightforward ultrasonic treatment method to precisely synthesize Ti_3_C_2_T_x_ nanosheets with adjustable sizes, and further conducted an in-depth investigation into the specific impact of nanosheet size on their supercapacitive performance. Experimental results indicate that, compared to larger nanosheets, smaller Ti_3_C_2_T_x_ nanosheets exhibit superior electrochemical performance, with a specific capacitance value as high as 658 F/g. This performance enhancement is primarily attributed to the unique advantages of smaller nanosheets. Specifically, their reduced size not only provides more ample space for the insertion of a large number of H^+^ ions but also significantly facilitates the penetration and distribution of water molecules within the Ti_3_C_2_T_x_ layers. These water molecules confined within the Ti_3_C_2_T_x_ layers play a crucial role in the charge storage mechanism of Ti_3_C_2_T_x_. They effectively activate the redox properties of titanium, making the charge storage process more efficient, and enable rapid charge compensation through the high diffusivity of protons, thereby further enhancing the charge/discharge rate and cycle stability of the supercapacitors. Additionally, we also found that an increased relative content of -OH groups in smaller Ti_3_C_2_T_x_ positively influences the charge storage process. These additional -OH groups contribute to charge storage through intercalation pseudocapacitance, providing more active sites for charge accumulation, and thus further improving the overall performance of the supercapacitors.

## Data Availability

All the relevant data are included in this published article.

## Supplementary Materials

The following supporting information can be downloaded at: https://www.mdpi.com/article/10.3390/molecules30020241/s1, Figure S1: (a,b) Galvanostatic discharge curves of TCS-0 and TCS-3 at current densities ranging from 1 to 10 A g^−1^, (c,d) Cyclic voltammetry curves of TCS-0 and TCS-3 at scan rates ranging from 5 to 50 mV s^−1^; Figure S2: Capacitance contributions of surface-controlled and diffusion-controlled processes at different scan rates for TCS-0, TCS-1 and TCS-2. Table S1: Summary of the performance of supercapacitors based on MXene composite materials. References [41,42,43,44,45,46,47,48,49,50] are cited in the supplementary materials.
