# Supplementary material for: Size Engineering of Ti3C2Tx Nanosheets for Enhanced Supercapacitance Performance
Source: Molecules. 2025 Jan 9;30(2):241. doi: 10.3390/molecules30020241 (PMC11767968; doi:10.3390/molecules30020241)
Supplement: Supplementary file 1 [file molecules-30-00241-s001.zip › molecules-3355994-supplementary.pdf]

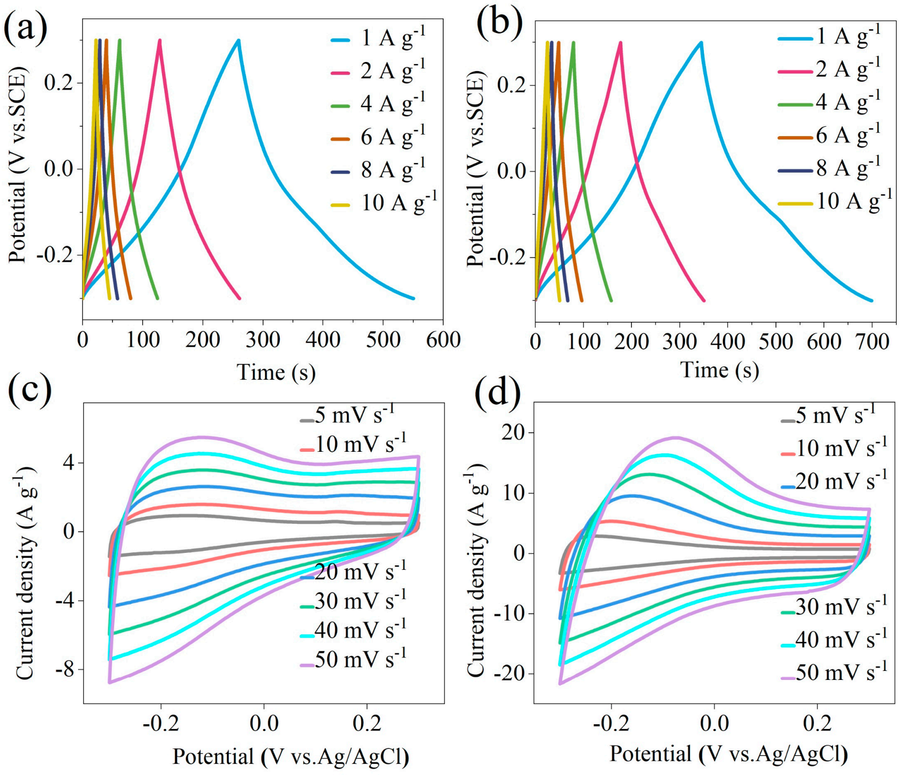

Figure S1. (a), (b) Galvanostatic discharge curves of TCS-0 and TCS-3 at current densities ranging from 1 to 10 A g^{-1}, (c), (d) Cyclic voltammetry curves of TCS-0 and TCS-3 at scan rates ranging from 5 to 50 mV s^{-1}.

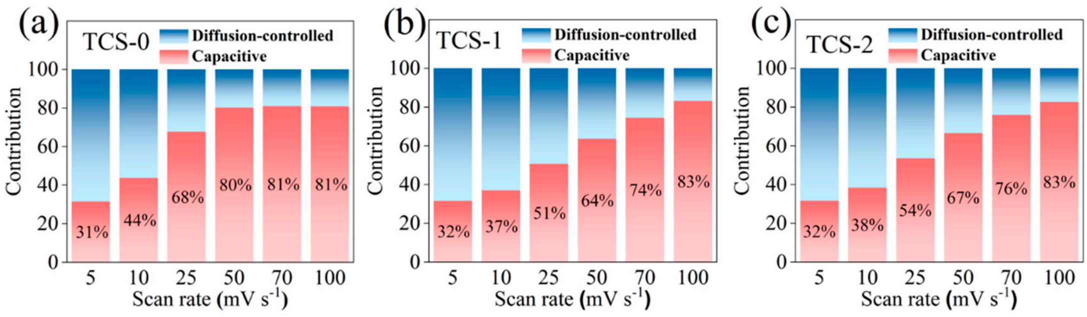

Figure S2. Capacitance contributions of surface-controlled and diffusion-controlled processes at different scan rates for TCS-0, TCS-1 and TCS-2.

Table S1. Summary of the performance of supercapacitors based on MXene composite materials.					
Material	Specific capacity	Electrolyte	Capacity retention(%)	Cycles	Ref.
MXene/PEA-derived HCNFs	350 F g^{-1} at 1 A g^{-1}	6 M KOH	93	3000	[1]
$Ti_3C_2T_x$/CDs	984.5 F cm^{-3} at 2 mV s^{-1}	1 M H_2SO_4	94.6	10000	[2]
$Ti_3C_2T_x$/CDs	1244.6 F cm^{-3} at 2 mV s^{-1}	3 M H_2SO_4	93.5	30000	[3]
$Ti_3C_2T_x$/CDs	144 F g^{-1} at 0.1 A g^{-1}	10 mM NaCl	101.1	10000	[4]
MXene/GDY-NTs	337.4 F g^{-1} at 2 A g^{-1}	1 M H_2SO_4	88.2	10000	[5]
4-amino-TEMPO/ MXene	446.9 F g^{-1} at 5 mV s^{-1}	1 M H_2SO_4	95.8	10000	[6]
MXene/polypyrrole	563.8 F g^{-1} at 0.5 A g^{-1}	1 M H_2SO_4	79.5	6000	[7]
Co-TCPP/MXene	1591.7 mF cm^{-2} at 1 mA cm^{-2}	1 M H_2SO_4	99.81	8000	[8]
MXene/CNTs@Ni	990.8 F cm^{-3}at 1 A g^{-1}	1 M H_2SO_4	/	/	[9]
MXene/PZS	380 F g^{-1} at 2 mV s^{-1}	1 M H_2SO_4	/	/	[10]
This work	658 F g^{-1}at 1 A g^{-1}	3 M H_2SO_4	100	20000	/

1. Kwon, Y. S.; Lee, J. S.; Hwang, G. H.; Jeong, Y. G., Hybrid Carbon Nanofibers Derived from MXene Nanosheets and Aromatic Poly(ether amide) for Self-Standing Electrochemical Energy Storage Materials. *Macromolecular Materials and Engineering*. **2022,** *307*, 2100877.
2. Li, L.; Wu, S.; Wu, K.; Zhou, H.; Li, Y.; Guo, M.; Qu, L.; Zhou, Y., Carbon Dot-Regulated 2D MXene Films with High Volumetric Capacitance. *Industrial & Engineering Chemistry Research*. **2020,** *59*, 13969-13978.
3. Xu, H.; Liu, Y. H.; Wang, Z. L.; Shi, K.; Zhang, B.; Yang, Y. B., General contact response of single-axle two-mass test vehicles for scanning bridge frequencies considering suspension effect. *Engineering Structures*. **2022,** *270*, 114880.
4. Tan, Z.; Wang, W.; Zhu, M.; Liu, Y.; Yang, Y.; Ji, X.; He, Z., Ti3C2Tx MXene@carbon dots hybrid microflowers as a binder-free electrode material toward high capacity capacitive deionization. *Desalination*. **2023,** *548*, 116267.
5. Wang, Y.; Chen, N.; Liu, Y.; Zhou, X.; Pu, B.; Qing, Y.; Zhang, M.; Jiang, X.; Huang, J.; Tang, Q.; Zhou, B.; Yang, W., MXene/Graphdiyne nanotube composite films for Free-Standing and flexible Solid-State supercapacitor. *Chemical Engineering Journal*. **2022,** *450*, 138398.
6. Chen, B.; Lu, Z.; Feng, S.; Zhou, Z.; Lu, C., Redox-Active Nitroxide Radicals Grafted onto MXene: Boosting Energy Storage via Improved Charge Transfer and Surface Capacitance. *ACS Energy Letters*. **2023,** *8*, 1096-1106.
7. Luo, W.; Sun, Y.; Han, Y.; Ding, J.; Li, T.; Hou, C.; Ma, Y., Flexible Ti3C2Tx MXene/polypyrrole composite films for high-performance all-solid asymmetric supercapacitors. *Electrochimica Acta*. **2023,** *441*, 141818.
8. Zhang, S.; Huang, Y.; Wang, J.; Zhang, S.; Han, X.; Sun, X., Co-TCPP/MXene film electrodes and patterned interdigital electrodes with high performance for flexible all-solid supercapacitors. *Journal of Energy Storage*. **2023,** *70*, 107973.
9. Li, S.; Zhang, Q.; Liu, L.; Wang, J.; Zhang, L.; Shi, M.; Chen, X., Ultra-stable sandwich shaped flexible MXene/CNT@Ni films for high performance supercapacitor. *Journal of Alloys and Compounds*. **2023,** *941*, 168963.
10. Li, L.; Niu, H.; Robertson, J.; Jiang, Z.; Guo, Y.; Kuai, C., Cyclocrosslinked polyphosphazene modified MXene as aqueous supercapacitor. *Electrochimica Acta*. **2023,** *439*, 141574.
